# The Knowledge, Attitudes and Practices of Practice Nurses in the Provision of Medication Abortion: A Cross‐Sectional Survey

**DOI:** 10.1111/jan.17051

**Published:** 2025-05-15

**Authors:** Sharon James, Satish Melwani, Stella May Gwini, Kirsten I. Black, Angela Taft, Deborah Bateson, Wendy V. Norman, Danielle Mazza

**Affiliations:** ^1^ SPHERE, NHMRC Centre of Research Excellence, Department of General Practice, School of Public Health and Preventive Medicine Monash University Melbourne Australia; ^2^ Monash Centre for Occupational & Environmental Health, School of Public Health and Preventive Medicine Monash University Melbourne Australia; ^3^ Specialty of Obstetrics, Gynaecology and Neonatology, Faculty of Medicine and Health University of Sydney Sydney Australia; ^4^ Judith Lumley Centre, School of Nursing and Midwifery LaTrobe University Melbourne Australia; ^5^ Faculty of Medicine and Health University of Sydney Sydney Australia; ^6^ Department of Family Practice University of British Columbia Vancouver Canada

**Keywords:** abortion, general practice, nursing, primary care, survey, women's health

## Abstract

**Aim:**

To examine practice nurse knowledge, attitudes, and practices about medication abortion in Australia.

**Design:**

Cross‐sectional survey.

**Methods:**

A national online survey was conducted from July to December 2021. Nurses working in general practice were recruited using convenience sampling. Data collected included demographics, knowledge, attitudes, and practices in abortion care. Analyses used included descriptive statistics and Poisson regression.

**Results:**

From 489 responses, knowledge about medication abortion, its provision, and efficacy was low. Although many respondents felt it was acceptable to assist in medication abortion, few indicated involvement. Those with advanced qualifications had greater perceived knowledge of abortion counselling. Respondent involvement in medication abortion was more likely if they had worked in general practice for a long time, their primary place of work was outside of general practice, or had advanced nursing qualifications.

**Conclusions:**

Given their role in the community, there is an opportunity to better utilise practice nurses for abortion care. Incorporation of abortion into the nursing curriculum and routine practice, including supportive funding mechanisms for care, is needed.

**Implications:**

Low knowledge and a lack of practice nurses providing abortion services adversely impact patient access.

**Impact:**

- Practice nurse provision of medication abortion has not yet been optimised.
- While practice nurses reported acceptability to provide abortion care, this could be enhanced with funding, education, and service normalisation.
- These results will inform policy makers, educators, patients, general practices, and nurses to support patient access to abortion care.
- Incorporating abortion care into nursing curriculum and practice will support women's access to these services.

**Reporting Method:**

CHERRIES guideline.

**Patient or Public Contribution:**

Professional groups, family planning organisations, industry, and government grant partners supported the study's recruitment.

**Trial Registration:**

ACTRN12622000655741

**Summary**

- What does this paper contribute to the wider global community?
  ○ Better practice nurse utilisation is required to enhance women's access to abortion care.
  ○ Incorporating abortion care into nursing curriculum and practice will support women’s access to these services.

## 1 Introduction

Universal access to sexual and reproductive health (SRH) services, such as pregnancy planning, and the incorporation of reproductive health into national policies are identified aims of the World Health Organization's Sustainable Development Goals (SDGs) (World Health Organization 2022). However, international progress in achieving the SDGs in relation to women's^1^ SRH has stagnated or worsened due to issues such as the COVID‐19 pandemic and/or criminalisation of abortion in some countries (World Health Organization 2022). This is at a time when international estimates show nearly 48% of pregnancies are unintended and 61% of these end in abortion (Bearak et al. 2020).

In the Australian context, unintended pregnancy impacts 26% of women, with 30% of these ending in abortion (Taft et al. 2018). The National Women's Health Strategy 2020–2030, together with a recent parliamentary inquiry, has recognised the need for equitable access to abortion and contraceptive services (Australian Government Department of Health 2018; Commonwealth of Australia 2023). The issue of ‘abortion deserts’, areas where there are no abortion providers, are common for rural and remote areas (Australian Government Department of Health 2018; Commonwealth of Australia 2023). In 2019, about 30% of women lived in an area where medication abortion (MA) had not been prescribed (Subasinghe et al. 2021). While abortion provision in Australia is legal, each state and territory have its own legislation such as who can conduct an abortion and two doctor approvals after a gestational limit has been reached (MSI Australia 2024). In addition, issues such as stigma and the historical mandated training requirements to prescribe MA meant that few (11%) Australian doctors provided these services (Australian Government Department of Health and Aged Care 2022; Commonwealth of Australia 2023; MS Health 2022). The lack of abortion providers in rural and remote areas could be addressed by supporting the training and workforce expansion of health care professionals who are able to provide this care (Commonwealth of Australia 2023).

## 2 Background

MA (also known as early medical abortion or medical termination of pregnancy) is licensed for use in Australia as a composite pack of mifepristone and misoprostol (sold as MS‐2 Step) up to 9 weeks' gestation. This service is largely provided in primary health care (PHC) settings by general practitioners (GPs) through general practices (also known as primary care) or through sexual health or family planning services (Subasinghe et al. 2021). However medical workforce shortages, particularly in rural areas, as well as stigma, isolation, costs and lack of clear referral pathways impact women's access to SRH services (Commonwealth of Australia 2023; Dawson et al. 2017). Strengthening nursing training roles could improve abortion access and expand patients' choice of health professionals who could provide this care. This approach would also be in line with international and Australian recommendations for nurses to be utilised in the provision of abortion and contraception services (Commonwealth of Australia 2023; World Health Organization 2022).

Practice nurses (PNs), who work in general practice settings, can form part of this solution. PNs require a licence/registration to practice with preparation as a diploma‐qualified Enrolled Nurse, baccalaureate‐prepared (or equivalent) Registered Nurse, or as a Registered Nurse with a Master's degree, clinical experience and endorsement as a Nurse Practitioner (Australian Government Department of Health and Aged Care 2023a). There are approximately 14,360 PNs in Australia and 92% of general practices employ a PN (Australian Government Department of Health and Aged Care 2022; The Royal Australian College of General Practitioners 2023). Most nursing care provided in general practice is funded as clinical encounters undertaken on behalf of the GP and is paid by the Australian government and/or patient (Freund et al. 2015). Practices are also offered block payments for the employment of health professionals to support clinical activities, which include PNs (Freund et al. 2015).

Internationally and in Australia, PN practice includes triage, prevention and health promotion, care coordination, treatment, acute and ongoing care (Australian Primary Health Care Nurses Association, n.d.; Health Education England 2017; Mid‐Central District Health Board and New Zealand Nurses Organisation Tōpūtanga Tapuhi Kaitaki O Aotearoa and the New Zealand College of Primary Health Care Nurses 2014 (Updated 2019)). Examples of PN areas of practice within the SRH context include cervical screening, sexual health promotion, sexually transmitted infection screening and follow‐up (Horwood et al. 2020; Mills et al. 2012; Wood et al. 2021). In other Australian PHC settings such as sexual and community health clinics, MA care by nurses has been established (Cashman et al. 2021; Tomnay et al. 2018). In the international context, abortion care is provided by nurses (Sium et al. 2025; Stirling‐Cameron et al. 2025) and has been shown to increase patient access to these services (Sium et al. 2025). However, PN roles and involvement in MA care in Australia are unclear and could provide a solution in areas where there are limited or no abortion providers.

## 3 The Study

### 3.1 Aim and Research Question

We aimed to describe the knowledge, attitudes and practices (KAP) of Australian PNs in relation to the provision of MA care to answer the research question ‘What are Australian PNs' knowledge, attitudes and practices about MA?’

### 3.2 Objectives

This study was part of the Australian Contraception and Abortion Primary Care Practitioner Support (AusCAPPS) Network (ACTRN12622000655741) (Mazza et al. 2022). The AusCAPPS Network is a national multidisciplinary online community of practice developed to improve women's access to long‐acting reversible contraception (LARC) and MA by supporting PNs, GPs, and pharmacists to provide these services in Australian primary care (Mazza et al. 2022).

## 4 Methods

### 4.1 Design

This survey is part of a larger survey with PNs, GPs, and pharmacists about their knowledge, attitudes, and practices in relation to long‐acting reversible contraception and MA. We used the CHERRIES guideline to support the reporting of survey results (File S1) (Eysenbach 2004).

### 4.2 Study Setting and Sample

We invited PNs working in Australian general practice to participate. A sample of approximately 500 PNs was sought (where the estimated population was 14,360 PNs (Australian Government Department of Health and Aged Care 2022)) to estimate the number and proportion of respondents with key demographic characteristics and MA knowledge, attitudes and practices with a precision of ±5% and assuming 50% of PNs were knowledgeable about MA. Using convenience sampling, survey recruitment included e‐mails and physical mail‐outs using a purchased health professional address list from the Australasian Medical Publishing Company, and dissemination of recruitment material through trial partner organisations, including the Australian Primary Health Care Nurses Association. Social media platforms (LinkedIn, X and Facebook groups such as the Australian General Practice Nurses Network) were also used for recruitment.

### 4.3 Inclusion and/or Exclusion Criteria

Clinician registration with the Australian Health Practitioner Regulation Agency (AHPRA) was a requirement for survey response inclusion. Given the volume of data, this paper only includes PN responses about MA.

### 4.4 Instrument

This online cross‐sectional survey was based on previous surveys about LARC and MA care (Jones et al. 2017; Mazza et al. 2016; Norman et al. 2016) and was informed and piloted by expert clinicians and academics in women's health and primary care. The final survey consisted of a maximum of 50 questions over nine pages in the English language. Questions could be revisited by study participants prior to submission. The survey took an estimated 10–15 min to complete (File S2).

Both closed and open‐ended questions formed part of the survey design. Demographics including age, identifying gender, location and number of years practising were included. Mandatory questions (as indicated in File S2), such as ‘Does your practice currently provide termination of pregnancy services? ‘were asked. Branching logic questions were adopted to further clarify or provide detail about the participant's previous selection. For example, if the participant's practice provided MA services, they were asked ‘Does your practice offer medical abortion services via telemedicine?’. Other questions collected nominal and ordinal data, including the use of 3‐point (agree, disagree, neither or yes, no, unsure) and 5‐point (strongly disagree to strongly agree, with ‘not applicable’ as an option) Likert scales. Free‐text responses were offered to provide detail on fields where participants had selected ‘other’ responses, such as ‘Who is aware of your work as a medical abortion provider?’. Following survey completion, we also invited respondents to join the AusCAPPS Network.

### 4.5 Data Collection

We launched the survey in July 2021 and recruitment concluded in December 2021 when the desired sample size was reached. All survey responses were captured and securely stored using REDCap (Research Electronic Data Capture), which was hosted and managed by Helix (Monash University) (Harris et al. 2009; Harris et al. 2019). Data collected from the survey were exported to Microsoft Excel for cleaning. On successful completion of the survey, we offered respondents a reimbursement of a $AUD40 e‐gift card for their time.

Given the risks associated with public surveys and financial reimbursement (e.g., bots and respondent misrepresentation), we used a verification process to mitigate survey fraud (Pratt‐Chapman et al. 2021). This included the matching of AHPRA registration to the participant's name and a combination of human‐ and computer‐based tools, such as a honeypot question only identifiable by computer, reCAPTCHA, checking for illogical or duplicate responses, and minimum timeframe screening of 5 min for survey completion, which was determined from piloting. Cookies and IP addresses were not recorded by the survey capture software.

### 4.6 Data Analysis

Descriptive statistical analysis, including counts, proportions as well as Poisson regression, were undertaken on STATA Statistical Software (version 18) (StataCorp 2023). The remoteness and population size of the participants' location were analysed using the Modified Monash Model (MMM) (Australian Government Department of Health and Aged Care 2021) and the participant's postcode. The demographic characteristics as well as knowledge, attitudes and practices of PNs about MA (e.g., true/false/unsure) were calculated as frequencies with percentages.

We used Poisson regression, with robust error sandwich estimators, to test for the association between respondent demographics (i.e., age, rurality and primary place of work as independent variables) and their degree of engagement in MA with their knowledge, attitudes, and practices (as dependent variables). Explanatory variables were chosen based on the research question and literature about clinician provision and patient access to MA care. Knowledge, attitudes, and practice questions with enough variability (of no more than 80% of PNs with one response) were calculated. The Poisson regression model was used in this context as an effect estimate (referred to as descriptive modelling by Shmueli (2010)) for the comparison of knowledge levels across participant subgroups adjusting for possible confounders, as opposed to its use as a predictive model. Hence, goodness of fit statistics were not reported. A Poisson regression model, which is often used for count data but in this case was used for binary data, was chosen in these analyses as opposed to Logistic regression because it provides better estimates of risk ratios and is easier to interpret (Barros and Hirakata 2003).

The results were reported as risk ratios (RR) together with the 95% confidence interval (CI). Given the low numbers of PNs involved in abortion care, we combined the categories of ‘some involvement’ and ‘led’ involvement to analyse the degree of involvement in MA. All questions where ‘other’ responses were indicated were grouped and analysed by key themes. Only those respondents who had completed all mandatory questions were included in the analysis. Given the variety and progressive nature of fraud mitigation strategies used, analysis on missing data beyond CHERRIES requirements was not undertaken (Eysenbach 2004).

### 4.7 Ethical Considerations

The study was approved by Monash University Human Research Ethics Committee (No. 28002). An explanatory statement was available for participants to read before survey commencement. Consent was implied by survey completion. All confidential data were accessible only by the research team, stored on a password‐protected file within a Monash University drive as per institution policy.

## 5 Results

### 5.1 Response Characteristics

**FIGURE 1:**
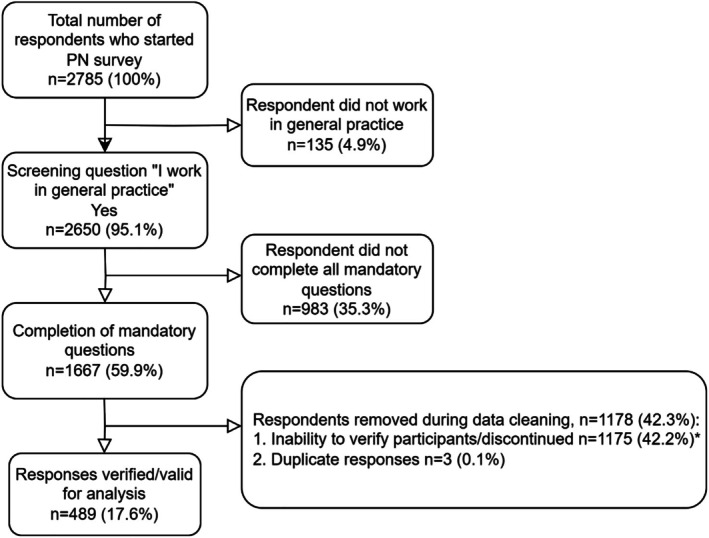
Response flow diagram. * Differentiation between discontinued surveys from some fraudulent responses may not be possible as respondents may have discontinued the survey at differing stages. In addition, the honeypot question was implemented as part of the verification process after survey launch and positioned prior to the respondent indicating their professional group.

From 2785 responses, 489 were valid. A response was counted if a respondent had answered the demographic fields, all mandatory questions and their response fulfilled the verification procedure. Responses that were excluded were due to the respondent not working in general practice (*n* = 135), an incomplete survey (*n* = 983), an inability to verify survey respondent as a nurse (*n* = 1175), or provision of duplicate responses (*n* = 3) (Figure 1). The calculation of unique site visits for the PN survey was not possible as a single survey link was used as a landing page to start the survey for all professional groups. Therefore, those who selected the option PN were counted as unique survey visitors. The participation rates at each stage of the response flow are found in Figure 1. Calculated from all users who completed the first survey question and all users who completed the final mandatory question ‘I feel confident to assist with medical abortions’ (*n* = 1828), the completion rate was 68.9%.

Survey respondents were mainly women working in metropolitan areas (Table 1). While age was evenly distributed across categories, there were slightly more respondents in the 50–54 years age group (14.1%). Most respondents were registered nurses (70.1%), had mainly worked in a general practice small business setting (93.1%) and had spent 0–4 years working in primary care (34.4%). Those who identified advanced practice qualifications were registered nurses (advanced practice) (14.5%) and nurse practitioners (7.2%). Of those who indicated a secondary place of work, they mainly worked in ‘other’ settings, such as hospital or community health (51.4%) or other general practice (45.8%). Few participants undertake consultations in a language other than English (10.6%) (e.g., Cantonese, Mandarin or Vietnamese). These demographic characteristics have similarity to national workforce data where PHC nurses have an average age of 51 years, predominantly practice in metropolitan areas (59%) and most are registered nurses (79%) (Australian Primary Health Care Nurses Association 2023).

**TABLE 1:** Demographic characteristics of practice nurses (*N* = 489).

	% (*n*)
Age	
18–24	4.7 (*23*)
25–29	10.4 (*51*)
30–34	11.3 (*55*)
35–39	12.7 (*62*)
40–44	10.6 (*52*)
45–49	10.8 (*53*)
50–54	14.1 (*69*)
55–59	12.3 (*60*)
60–64	10.2 (*50*)
65+	2.9 (*14*)
Gender	
Woman	97.6 (*477*)
Man	1.8 (*9*)
Nonbinary	0.4 (*2*)
Prefer not to answer	0.2 (*1*)
Rurality	
1 (Metropolitan)	63.3 (*307*)
2–5 (Regional/Rural)	34.9 (*169*)
6–7 (Remote/Very Remote)	1.9 (*9*)
Primary place of work	
General practice	93.1 (*455*)
Women's health service	1.6 (*8*)
Family planning organisation	0.6 (*3*)
Refugee health	0.6 (*3*)
Other	4.1 (*20*)
Years worked in general practice	
0–4	34.4 (*168*)
5–9	27.8 (*136*)
10–14	18.8 (*92*)
15–19	8.6 (*42*)
20–24	5.3 (*26*)
25–29	2.9 (*14*)
30+	2.3 (*11*)
General practice type	
Small business	66.9 (*327*)
Corporate chain	16.0 (*78*)
Other, e.g., Aboriginal Health Corporation, Community Health Service	9.8 (*48*)
Not stated	7.4 (*36*)
Qualifications held (*multiple choices allowed*)	
Enrolled nurse	9.4 (*46*)
Registered nurse	70.1 (*343*)
Registered nurse (advanced practice)	14.5 (*71*)
Nurse practitioner	7.2 (*35*)
Other, e.g., Midwife, Child and Family Health Nurse	3.9 (*19*)
Consults in language other than English	
No	89.4 (*437*)
Yes	10.6 (*52*)

### 5.2 PN MA Knowledge

Knowledge about gestational limits, provision, and efficacy of MA was low among the survey respondents. More than half (53.6%) were not aware or were unsure of the 9‐week gestational limit for the provision of MA. While 46.4% of respondents recognised the comparable efficacy of MA to the procedural option (Table 2), respondents were less likely to report a correct response about MA efficacy compared to procedural abortion if they worked in general practice as a primary place of work (Table 3). However, a correct response was more likely among respondents aged > 50 years, with more than 5 years worked in general practice and among those with the highest qualifications (advanced practice registered nurse and nurse practitioner) (Table 3).

**TABLE 2:** Practice nurses' knowledge and attitudes about MA.

	Row % (*n*)	Row % (*n*)	Row % (*n*)	
Knowledge statements	True	False	Unsure	
In Australia, MA is registered for use up to 9 weeks (63 days) gestation in all states—True	46.4 (*227*)	9.6 (*47*)	44.0 (*215*)	
Efficacy of MA is similar to that of surgical abortion—True	46.4 (*227*)	13.7 (*67*)	39.9 (*195*)	
Misoprostol is administered before mifepristone—False	11.7 (*57*)	20.2 (*99*)	68.1 (*333*)	
MA medicines can be self‐administered at home—True	66.7 (*326*)	7.2 (*35*)	26.2 (*128*)	
Attitudes statement	Agree	Disagree	Neither	
I have the knowledge to counsel women about the process of MA	15.5 (*76*)	69.1 (*338*)	15.4 (*75*)	
I feel confident in managing MAs	9.0 (*44*)	75.5 (*369*)	15.5 (*76*)	
I feel confident to assist in MA provision	23.1 (*113*)	62.8 (*307*)	14.1 (*69*)	
It is acceptable for nurse/nurse practitioners to assist with MA	71.0 (*347*)	8.8 (*43*)	20.2 (*99*)	
I think women need to know more about the availability of MA	89.4 (*437*)	3.5 (*17*)	7.1 (*35*)	
Attitudes statement	(Strongly) Disagree	Neither agree nor disagree	(Strongly) Agree	Not applicable
Media take a balanced view on MA(*N* = 489)	54.8 (*268*)	30.5 (*149*)	8.8 (*43*)	5.9 (*29*)
I feel discriminated against by other healthcare professionals because of my decision to provide MA services (*N* = 116)	39.7 (*46*)	24.1 (*28*)	6.0 (*7*)	30.2 (*35*)
I am proud that I work in abortion care (*N* = 116)	5.2 (*6*)	20.7 (*24*)	41.4 (*48*)	32.8 (*38*)
I feel connected to others who do this work (*N* = 116)	11.2 (*13*)	32.8 (*38*)	34.5 (*40*)	21.6 (*25*)
I feel that society appreciates the work I do in abortion care (*N* = 116)	12.9 (*15*)	27.6 (*32*)	25.0 (*29*)	34.5 (*40*)
I am afraid that if I tell people I work in abortion care I could put myself or my loved ones at risk of harassment/violence (*N* = 116)	35.3 (*41*)	20.7 (*24*)	12.9 (*15*)	31.0 (*36*)
I have never experienced harassment and/or violence as a result of working in abortion care (*N* = 116)	8.6 (*10*)	8.6 (*10*)	54.3 (*63*)	28.5 (33)

**TABLE 3:** Relationship between respondent characteristics and MA knowledge.

Characteristic	Knowledge statements							
	MA is registered for use up to 9 weeks (True)		MA efficacy is similar to procedural abortion (True)		Misoprostol is administered before mifepristone (False)		MA medicines can be self‐administered at home (True)	
	Row % (*n*)	RRR (95% CI)	Row % (*n*)	RRR (95% CI)	Row % (*n*)	RRR (95% CI)	Row % (*n*)	RRR (95% CI)
Age (years)								
< 30	50.0 (*37*)	Ref	33.8 (*25*)	Ref	23.0 (*17*)	Ref	48.7 (*36*)	Ref
30–39	47.9 (*56*)	0.96 (0.71–1.29)	39.3 (*46*)	1.16 (0.79–1.72)	16.2 (*19*)	0.71 (0.39–1.27)	68.4 (*80*)	**1.41 (1.08–1.83)**
40–49	40.0 (*42*)	0.80 (0.58–1.11)	46.7 (*49*)	1.38 (0.95–2.01)	20.0 (*21*)	0.87 (0.49–1.53)	69.5 (*73*)	**1.43 (1.09–1.86)**
50–59	48.8 (*63*)	0.98 (0.73–1.30)	53.5 (*69*)	**1.58 (1.11–2.26)**	25.6 (*33*)	1.11 (0.67–1.86)	71.3 (*92*)	**1.47 (1.13–1.90)**
60+	45.3 (*29*)	0.91 (0.64–1.29)	59.4 (*38*)	**1.76 (1.20–2.57)**	14.1 (*9*)	0.61 (0.29–1.28)	70.3 (*45*)	**1.45 (1.09–1.92)**
Rurality								
Other	46.1 (*82*)	Ref	46.1 (*82*)	Ref	21.4 (*38*)	Ref	68.5 (*122*)	Ref
Metropolitan	46.6 (*143*)	1.01 (0.83–1.23)	47.2 (*145*)	1.02 (0.84–1.25)	19.9 (*61*)	0.93 (0.65–1.34)	65.2 (*200*)	0.95 (0.84–1.08)
Primary place of practice								
Other	52.9 (*18*)	Ref	61.8 (*21*)	Ref	29.4 (*10*)	Ref	67.7 (*23*)	Ref
General practice	45.9 (*209*)	0.87 (0.62–1.21)	45.3 (*206*)	**0.73 (0.55–0.97)**	19.6 (*89*)	0.67 (0.38–1.17)	66.6 (*303*)	0.98 (0.77–1.25)
Years worked in general practice								
< 5	43.4 (73)	Ref	32.7 (55)	Ref	21.4 (36)	Ref	58.9 (99)	Ref
5–9	37.8 (65)	1.10 (0.86–1.41)	48.5 (66)	**1.48 (1.13–1.96)**	21.3 (29)	0.99 (0.64–1.53)	69.1 (94)	1.17 (0.99–1.39)
10–14	52.2 (48)	1.20 (0.93–1.56)	56.2 (52)	**1.73 (1.30–2.29)**	17.4 (16)	0.81 (0.48–1.38)	71.7 (66)	**1.22 (1.02–1.46)**
15+	44.1 (41)	1.01 (0.76–1.35)	58.1 (54)	**1.77 (1.34–2.34)**	19.3 (18)	0.90 (0.54–1.50)	72.0 (67)	**1.22 (1.02–1.46)**
Highest qualifications held								
Enrolled nurse (*N* = 46)	41.3 (*19*)	0.93 (0.65–1.35)	34.8 (*16*)	0.80 (0.53–1.21)	10.9 (*5*)	0.61 (0.26–1.43)	58.7 (*27*)	0.94 (0.72–1.21)
Registered nurse (*N* = 340)	44.2 (*148*)	Ref	43.6 (*146*)	Ref	17.9 (*60*)	Ref	62.7 (*210*)	Ref
Registered nurse (advanced practice) (*N* = 76)	53.5 (*38*)	1.21 (0.95–1.55)	59.2 (*42*)	**1.36 (1.08–1.71)**	21.1 (*15*)	1.18 (0.71–1.95)	85.9 (*61*)	**1.37 (1.21–1.55)**
Nurse practitioner (*N* = 36)	57.1 (*20*)	1.29 (0.95–1.77)	60.0 (*21*)	**1.38 (1.02–1.85)**	54.3 (*19*)	**3.03 (2.07–4.44)**	77.1 (*27*)	**1.23 (1.01–1.50)**

*Note:* Bolded results indicate significant findings at *p* < 0.05.

Most respondents were unsure about the order of medication administration for a MA (68.1%), but those who had qualifications as a nurse practitioner were more likely to know the correct order of MA medicines administration (Table 3). Similarly, over two‐thirds (66.7%) of respondents knew that MA can be self‐administered at home; however, this knowledge was more likely based on > 10 years worked in general practice and highest qualifications held (advanced practice registered nurse and nurse practitioner).

### 5.3 PN Attitudes about MA

Most respondents (89.4%) agreed that it is important for women to know more about the availability of MA services and many (71.0%) agreed that it is acceptable for nurses/nurse practitioners to assist with MA provision (Table 2). Over a third disagreed that they felt discriminated against by other healthcare professionals because of their abortion care provision (39.7%) or that they were afraid to tell others about their abortion care provision, fearing to put themselves or their loved ones at risk of harassment/violence (35.3%). However, over half (54.8%) of respondents disagreed that the media takes a balanced view on MA (Table 2).

Most respondents indicated they did not or were unsure whether they had the knowledge to counsel women about abortion (84.6%), with nurses primarily working in general practice being more likely to disagree that they have this knowledge (Table 4). Registered nurses who indicated advanced practice or nurse practitioner qualifications were less likely to disagree with the statements regarding having perceived knowledge or confidence in managing MA, compared with registered nurses with no advanced training.

**TABLE 4:** Relationship between respondent characteristics and attitudes towards MA.

Characteristic	Disagreement with the following attitude statements					
	I have the knowledge to counsel women about the process of MA		I feel confident in managing MA		I feel confident to assist in MA provision	
	Row % (*n*)	RRR (95% CI)	Row % (*n*)	RRR (95% CI)	Row % (*n*)	RRR (95% CI)
Age						
< 30	71.6 (*53*)	Ref	78.4 (*58*)	Ref	71.6 (*53*)	Ref
30–39	74.4 (*87*)	1.04 (0.87–1.24)	78.6 (*92*)	1.00 (0.86–1.17)	65.0 (*76*)	0.91 (0.75–1.10)
40–49	73.3 (*77*)	1.02 (0.85–1.23)	81.0 (*85*)	1.03 (0.89–1.20)	63.8 (*67*)	0.89 (0.73–1.09)
50–59	65.1 (*84*)	0.91 (0.75–1.10)	70.5 (*91*)	0.90 (0.76–1.06)	62.0 (*80*)	0.87 (0.71–1.05)
60+	57.8 (*37*)	0.81 (0.63–1.04)	67.2 (*43*)	0.86 (0.70–1.06)	48.4 (*31*)	**0.68 (0.51–0.90)**
Rurality						
Other	66.9 (*119*)	Ref	77.0 (*137*)	Ref	64.6 (*115*)	Ref
Metropolitan	70.4 (*216*)	1.05 (0.93–1.19)	74.6 (*229*)	0.97 (0.87–1.08)	62.2 (*191*)	0.96 (0.84–1.11)
Primary place of practice						
Other	50.0 (*17*)	Ref	64.7 (*22*)	Ref	44.1 (*15*)	Ref
General practice	70.6 (*321*)	**1.41 (1.00–1.99)**	76.3 (*347*)	1.18 (0.91–1.52)	64.2 (*292*)	1.45 (0.99–2.14)
Years in general practice						
< 5	75.6 (127)	Ref	80.9 (136)	Ref	70.3 (118)	Ref
5–9	70.6 (96)	0.93 (0.81–1.07)	74.3 (101)	0.92 (0.81–1.04)	63.2 (86)	0.90 (0.76–1.06)
10–14	65.2 (60)	0.86 (0.73–1.02)	73.9 (68)	0.91 (0.79–1.05)	55.4 (51)	**0.79 (0.64–0.97)**
15+	59.1 (55)	**0.78 (0.64–0.94)**	68.8 (64)	**0.85 (0.73–0.99)**	55.9 (52)	**0.80 (0.65–0.98)**
Highest qualifications held						
Enrolled nurse (*N* = 46)	63.0 (*29*)	0.83 (0.66–1.05)	69.6 (*32*)	0.87 (0.71–1.06)	54.4 (*25*)	0.78 (0.60–1.03)
Registered nurse (*N* = 340)	75.5 (*253*)	Ref	80.3 (*269*)	Ref	69.3 (*232*)	Ref
Registered nurse (advanced practice) (*N* = 76)	54.9 (*39*)	**0.73 (0.58–0.91)**	62.0 (*44*)	**0.77 (0.64–0.93)**	45.1 (*32*)	**0.65 (0.50–0.85)**
Nurse practitioner (*N* = 36)	45.7 (*16*)	**0.61 (0.42–0.87)**	62.9 (*22*)	0.78 (0.60–1.02)	45.7 (*16*)	**0.66 (0.46–0.95)**

*Note:* Bolded results indicate significant findings at *p* < 0.05.

Only 23.1% of respondents agreed about having the confidence to assist in MA. Clinically relevant factors that were found to be negatively associated with respondents disagreeing they had the confidence required to assist in MA were years worked in general practice and having higher qualifications (advanced practice registered nurse and nurse practitioner). Factors that would dissuade a respondent from counselling an eligible patient on MA included not feeling they have the knowledge (62.0%) and skills to assist (39.6%). Some of the factors listed by respondents in the ‘other’ category were knowledge, training, distance from hospital care (2.6%) or due to cost to clinic (2.2%) (Table 5).

**TABLE 5:** MA services provided by practice nurses.

	% (*n*)
**Practice provision of termination of pregnancy services**	
No	76.3 (*373*)
*Have someone to refer patients to*	
No	50.7 (*189*)
Yes	49.3 (*184*)
Yes, surgical abortion	1.0 (*5*)
Yes, medication and procedural abortion	2.3 (*11*)
Yes, MA	20.4 (*100*)
**Nurse/nurse practitioner involvement in MA services within their practice** (*N* = 116)	
No nurse involvement	60.4 (*67*)
Some nurse/nurse practitioner involvement	32.4 (*36*)
Nurse/nurse practitioner led/managed	7.2 (*8*)
**Do you offer MA services via telehealth?** (*N* = 111)	
No	76.6 (*85*)
Yes	23.4 (*26*)
**Who is aware of your work as a MA provider?** (*N* = 111)	
GPs in my practice	81.1 (*90*)
My practice manager/receptionist	61.3 (*68*)
Other nurses at my practice	61.3 (*68*)
Other local health professionals	40.5 (*45*)
A local pharmacist	36.9 (*41*)
A local GP	36.0 (*40*)
A local gynaecologist	21.6 (*24*)
My local emergency	17.1 (*19*)
The local radiology practice/s	13.5 (*15*)
The local pathology provider/s	12.6 (*14*)
Other	9.9 (*11*)
**Factors that would dissuade the nurses from counselling on MA to an eligible patient**	
I don't feel I have the knowledge to assist in the provision of a MA	63.4 (*310*)
I don't feel I have the skills to assist in the provision of a MA	40.5 (*198*)
Concerns about safety	8.6 (*42*)
I am afraid of the stigma associated with providing MA	8.6 (*42*)
Lack of referral options to specialists/hospitals if a complication occurs	8.4 (*41*)
Cost to patient	8.2 (*40*)
I am a conscientious objector to termination of pregnancy	5.3 (*26*)
Concerns about efficacy	4.5 (*22*)
Other	2.7 (*13*)
Cost to the clinic	2.3 (*11*)
Previous negative experience supporting a patient undergoing MA	2.7 (*13*)

**FIGURE 2:**
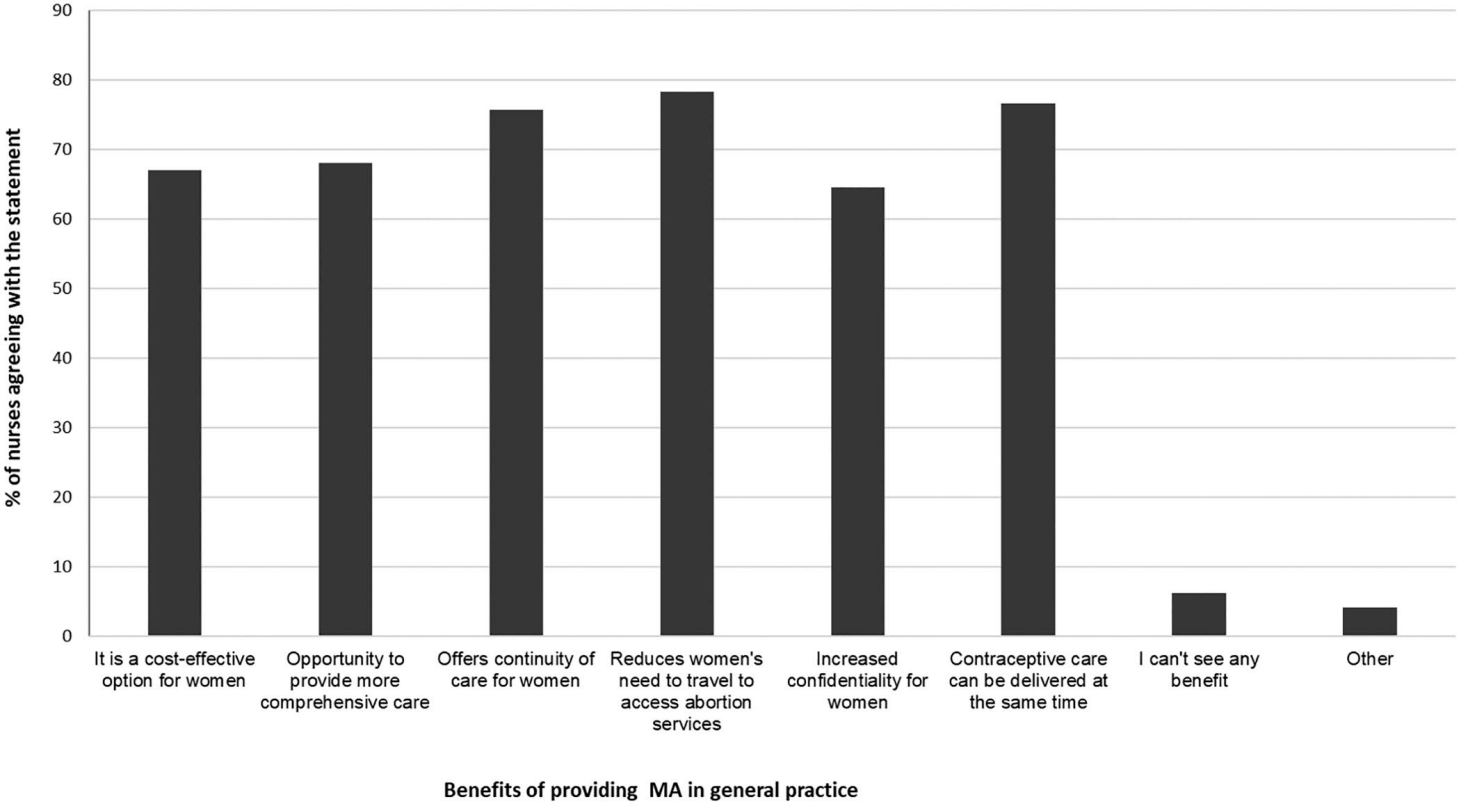
Practice nurses' attitudes about benefits to providing MA services in general practice.

Respondents indicated that the benefits of providing MA in general practice were mainly related to reducing women's need to travel to access abortion services (78.3%; *n* = 383), the ability to provide contraceptive care at the same time (76.7%; *n* = 375), as well as continuity of care for women (75.7%; *n* = 370). ‘Other’ responses included accessibility, flexibility, privacy, and trust (Figure 2).

### 5.4 PN MA Practices

Most respondents indicated that their practice did not offer abortion services (76.3%) (Table 6). Few respondents were involved in/managed MA services in their practice (8.6%), or offered these services by telehealth (5.3%) (Table 5). Of the PN respondents who indicated involvement in MA care, this mainly involved triage, counselling, organising pathology services and referrals, and follow‐up. Nurse involvement for abortion provision was more likely if the respondent's primary place of work was outside of general practice, if the respondent indicated > 15 years worked in general practice, and if they held qualifications as an advanced practice registered nurse or as a nurse practitioner (Table 6). For those who were working in abortion care, their coworkers within the same practice were most likely to know about their practice (46.2%) (Table 5). Reasons that respondents would be dissuaded from providing MA were largely associated with not having the knowledge (63.4%) and skills (40.5%) to provide this care.

**TABLE 6:** Relationship between respondent characteristics and involvement in MA.

Characteristics	Nurse involvement (Some and led/managed)	
	Row % (*n*)	RRR (95% CI)
Age		
< 30	25.0 (3)	0.78 (0.25–2.44)
30–39	32.0 (8)	Ref
40–49	32.1 (9)	1.00 (0.46–2.21)
50–59	57.1 (16)	1.79 (0.92–3.45)
60+	44.4 (8)	1.39 (0.64–3.01)
Rurality		
Other	43.3 (26)	Ref
Metropolitan	33.3 (16)	0.77 (0.47–1.26)
Primary place of practice		
Other	72.7 (8)	Ref
General practice	36.0 (36)	**0.50 (0.32–0.78)**
Years worked in general practice		
< 5	4.8 (8)	Ref
5–9	8.8 (12)	1.85 (0.78–4.41)
10–14	10.9 (10)	2.28 (0.93–5.59)
15+	15.1 (14)	**3.16 (1.38–7.26)**
Highest qualifications held		
Enrolled nurse (*N* = 8)	37.5 (3)	1.19 (0.46–3.10)
Registered nurse (*N* = 76)	31.6 (24)	Ref
Registered nurse (advanced practice) (*N* = 18)	55.6 (10)	**1.76 (1.03–2.99)**
Nurse practitioner (*N* = 9)	77.8 (7)	**2.46 (1.52–3.99)**

*Note:* Bolded results indicate significant findings at *p* < 0.05.

## 6 Discussion

This study aimed to describe the knowledge, attitudes and practices of Australian PNs in relation to the provision of MA care. We have identified limited involvement of Australian PNs in abortion care, exacerbated when general practice is the primary place of work. Knowledge gaps were identified regarding gestational limits, medication order and efficacy of MA. While respondents felt it was acceptable to provide abortion care, this was seriously limited by the knowledge and confidence to do so. Concern about discrimination and harassment when providing these services was low. However, concern about balanced media representation of abortion care is likely reflective of how respondents perceived this care may be interpreted by the community.

Internationally, issues such as the time‐critical nature of MA, the limited number of providers, distance, and geography present barriers to accessing abortion services (Hulme et al. 2015; Subasinghe et al. 2021). This study also indicates that a lack of PN capacity building, training opportunities, and practical experience creates further barriers to abortion service availability. Low respondent knowledge and confidence about abortion is likely reflected by the exposure needed in the undergraduate nursing curriculum to provide this care (Shi et al. 2025). A lack of emphasis on undergraduate PHC preparation and limited provision of abortions in hospital settings further perpetuates a lack of clinical knowledge and experience in abortion management (Commonwealth of Australia 2023; Murray‐Parahi et al. 2020). Yet, student nurses are interested in curricula preparing them to provide abortion care (King et al. 2025). Competing priorities within an undergraduate curriculum are likely to relegate abortion care to a specialty where knowledge is gained through advanced practice skills.

Few participants provided MA and fewer still were concerned about discrimination or harassment as a result of working in abortion care. Harassment experienced by clinicians providing this service is diffused where services have lower patient volume and safe access zones around abortion clinics (Ennis et al. 2023). Further integration of abortion care in the general practice setting alongside other clinical presentations would therefore support service provision. This can be offset where there are clinician concerns about public stigma when advertising abortion services, likely reflected in participant concerns about media reporting about abortion. However, to support patient access to services and normalise abortion as healthcare and a human right, destigmatisation of abortion is needed at a community level.

Results from this study indicate that PN involvement in abortion care is limited in the general practice setting, and less likely if their primary place of work is in the general practice setting. General practice offers opportunity for multidisciplinary and collaborative medical, nursing, and allied health team‐based care. However, current Australian funding mechanisms in this setting are aligned to the management of chronic conditions where PNs often provide care on behalf of the GP (Freund et al. 2015). This limits the professional autonomy and scope of practice for nurses to provide preventive SRH care. Nurses working in PHC settings have positively impacted population health initiatives, such as immunisations (e.g., child health and COVID‐19 programmes) and the management of chronic disease care (Australian Primary Health Care Nurses Association 2023; Health Education England 2017). With supportive policy and low to no patient cost arrangements, there is opportunity for PNs to make a similar impact in preventive SRH, including abortion care.

Most participants agreed that abortion care was an acceptable part of their role. Through leadership and role optimisation, abortion care as part of nursing scope of practice could be enabled to support equitable access for patients (Carson et al. 2022). While some literature identifies an expanded scope of nursing practice in abortion care that achieves similar effectiveness and safety as other providers, these services are also provided in an overregulated landscape in many countries (Mainey et al. 2020). In Australia, recent deregulation of MA to allow nurse practitioners and eligible midwives to prescribe MA (Australian Government Department of Health and Aged Care 2023b) still requires each state/territory to provide legislative approvals to go ahead as well as education and support for this expanded scope of practice. However, the normalisation of MA care for all nurses will support its provision as part of routine PHC and reduce stigma for patients and clinicians.

### 6.1 Strengths and Limitations

This study examined abortion provision by nurses through a national survey and therefore offers unique insights that can inform an evolving policy landscape regarding this care. There were a high number of fraudulent respondents in the survey, likely due to the nature of public surveys and the offer of e‐gift cards (Pratt‐Chapman et al. 2021). However, the combination of known human and electronic verification processes that were used supports data validity. In addition, the sample's demographics, such as gender, registration type, and years worked in general practice, are comparable to other PHC workforce data (Australian Primary Health Care Nurses Association 2023). Given the variety and progressive strategies used to mitigate survey fraud and ensure data integrity, it is not known whether incomplete surveys are a result of respondent difficulties completing particular survey questions. However, user testing was conducted with researchers and nurses to ensure face and content validity prior to the survey launch.

### 6.2 Recommendations for Further Research

To support improving the quality of and access to abortion care, further research could focus on the role of PNs in remote areas or the utility of conducting consultations in a language other than English. In addition, future research could examine PN educational pathways and alternative models of care to enhance the accessibility of MA services.

### 6.3 Implications for Policy and Practice

In the context of improving community access and overcoming medical workforce challenges, there is an opportunity to enhance PN knowledge and skills in abortion care as part of the undergraduate curriculum and continuing professional development. However, further capacity building, training opportunities, and practical experience are needed to enable PN knowledge and confidence to provide this care. Supportive policy and funding arrangements enabling training and clinical time to provide abortion care could enable PNs confidence to practice and enhance women's access to these services.

## 7 Conclusion

Our national survey of PNs found that most were open to participate in providing abortion care, but few had done so. The general practice setting as well as PNs' low knowledge and confidence to provide abortion services restrict patient access. Normalisation of abortion care as part of nursing education and routine practice would support its provision. However, supportive policy, funding, and training arrangements are needed to facilitate a shift to PN implementation of MA into practice.

## Author Contributions

Made substantial contributions to conception and design, or acquisition of data, or analysis and interpretation of data; Sharon James, Satish Melwani, Stella May Gwini, Kirsten I. Black, Deborah Bateson, Angela Taft, Wendy V. Norman and Danielle Mazza Involved in drafting the manuscript or revising it critically for important intellectual content; Sharon James, Satish Melwani, Stella May Gwini, Kirsten I. Black, Deborah Bateson, Angela Taft, Wendy V. Norman and Danielle Mazza Given final approval of the version to be published. Each author should have participated sufficiently in the work to take public responsibility for appropriate portions of the content; Sharon James, Satish Melwani, Stella May Gwini, Kirsten I. Black, Deborah Bateson, Angela Taft, Wendy V. Norman and Danielle Mazza Agreed to be accountable for all aspects of the work in ensuring that questions related to the accuracy or integrity of any part of the work are appropriately investigated and resolved; Sharon James, Danielle Mazza.

## Disclosure

Statistician: Dr. Gwini (Author 3).

## Conflicts of Interest

Sharon James is a Board Director of the Australian PHC Nurses Association. Deborah Bateson has provided clinical education about contraception for Mayne Pharma, Bayer and Besins but has not received personal remuneration for these services.

## Supporting information


**File S1.** Checklist for Reporting Results of Internet E‐Surveys (CHERRIES) (Eysenbach 2004).

**File S2.** Practice Nurse Knowledge, Attitudes and Practices Survey.

## Data Availability

The data are not publicly available due to privacy or ethical restrictions.

## Endnotes

^1^
People who do not identify as women may also require sexual and reproductive healthcare, including abortions.
