# Supplementary material for: The Knowledge, Attitudes and Practices of Practice Nurses in the Provision of Medication Abortion: A Cross‐Sectional Survey
Source: J Adv Nurs. 2025 May 15;82(3):2152–64. doi: 10.1111/jan.17051 (PMC12907591; doi:10.1111/jan.17051)
Supplement: Supplementary file 2 — File S2. Practice Nurse Knowledge, Attitudes and Practices Survey. [file JAN-82-2152-s001.docx]

**Supplementary file 2**

**Practice Nurse Knowledge, Attitudes and Practices Survey**

| **SECTION 1: DEMOGRAPHICS**  **How many years have you worked in general practice?** ______________*  **Primary practice post code**  ___________*  And other if applicable ________  **Gender***  Male or man  Female or woman  Non-binary  My gender identity isn't listed. I identify as: ______  Prefer not to answer  **Age (years)***  18-24  25-29  30-34  35-39  40-44  45-49  50-54  55-59  60-64  65+  **Please select the qualification(s) relevant to you:***  Enrolled Nurse  Registered Nurse  Registered Nurse (advanced practice)  Nurse Practitioner  Overseas trained  Other ______  **What is your primary place of work?***  ☐General Practice  ☐Family Planning Organisation  ☐Refugee Health  ☐Marie Stopes Australia  ☐Women’s Health Service  ☐Other ______  If yes to general practice  **Which of the following best describes the type of general practice in which you work?***  Small business  Corporate chain  Other ______  **What is your secondary place of work? ***  ☐General Practice  ☐Family Planning Organisation  ☐Refugee Health  ☐Marie Stopes Australia  ☐Women’s Health Service  ☐Other ______  If yes to general practice  **Which of the following best describes the type of general practice in which you work? ***  Small business  Corporate chain  Other ______  **Do you ever conduct your clinical consultations in a language other than English? ***  Yes (Please specify) ______  No |
| --- |
| **SECTION 2: PRACTICES**  *Please answers these questions in relation to general practice*  **Q1 Please answer the following questions about intrauterine devices (IUDs) and contraceptive implants in your practice?**   \|  \| **Intrauterine devices** \| **Contraceptive implants** \| \| --- \| --- \| --- \| \| **Q1a Do you insert and/or remove? *** \| I Insert and remove  I only insert  I only remove  Neither \| I Insert and remove  I only insert  I only remove  Neither \| \| **Q1b How many have you inserted in a typical month? ***  **(Please enter '0' if you do not insert)** \| _____ \| _______ \| \| **Q1c Have you received specific training** **in inserting or removing Intrauterine devices or implants? *** \| Yes  No  If yes, where?  Family Planning course  Other__________ \| Yes  No  If yes, where?  Family Planning course  Other__________ \| \| **Q1d** Where do your patients **typically have their insertions? Tick all that apply. *** \| I insert  Private gynaecologist  Public hospital  Family planning clinic  Another nurse/nurse practitioner in my practice  Another nurse/nurse practitioner in a local practice  GP in my practice  Another GP in a local practice  Other _________ \| I insert  Private gynaecologist  Public hospital  Family planning clinic  Another nurse/nurse practitioner in my practice  Another nurse/nurse practitioner in a local practice  GP in my practice  Another GP in a local practice  Other _________ \|   **Q2 How often would you initiate discussion about long-acting reversible contraceptives (e.g. intrauterine devices and contraceptive implants) in your contraceptive consultations? ***  Never  Rarely  Sometimes  Very often  Always |
| **Q3 Does your practice currently provide termination of pregnancy services? ***  Yes, medical abortion (go to Q4)  Yes, both medical abortion and surgical abortion (go to Q4)  Yes, surgical abortion (go to Q3a and then go to Q9)  No (go to Q3a and then go to Q9)  **Q3a Do you have any colleagues to whom you can refer for medical abortion if required? ***  Yes  No  **Q4 What involvement do nurse/ nurse practitioners have in medical abortion services within your practice? ***  Nurse/nurse practitioner led/managed  Some nurse/nurse practitioner involvement (please indicate this involvement) ______  No nurse involvement  **Q5 Does your practice offer medical abortion services via telemedicine?**  Yes  No  **Q6 After counselling for medical abortion, does your practice also discuss intrauterine device insertion? ***  Yes, by another nurse/nurse practitioner  Yes, by a GP  Yes, by myself  No  **Q7 After counselling for medical abortion, does your practice also discuss contraceptive implant insertion? ***  Yes, by another nurse/nurse practitioner  Yes, by a GP  Yes, by myself  No  **Q8 Who is aware of your work in medical abortion services? Please tick all that apply.**  A local pharmacist  A local GP  Other local health professionals  My practice manager/receptionist  GPs in my practice  Other practice nurses in my practice  The local radiology practice(s)  The local pathology provider(s)  A local gynaecologist  My local emergency department  Other ___________ |
| **SECTION 3: KNOWLEDGE**  **Q9 Please indicate your agreement with the following statements: ***  True  False  Unsure   1. Long-acting reversible contraceptives are less effective than the oral contraceptive pill at preventing pregnancy 2. Intrauterine devices are suitable for use in nulliparous women 3. Nurse/nurse practitioner’s view and advice can influence the type of contraception chosen by patients 4. Fertility can return rapidly after long-acting reversible contraceptive removal 5. In Australia medical abortion is registered for use up to 9 weeks (63 days) gestation in all states 6. Efficacy of medical abortion is similar to that of surgical abortion 7. Misoprostol is administered before mifepristone during a medical abortion 8. Medical abortion medicines can be self-administered at home   **Q10 What are the most common side effects from medical abortion? Please tick all that apply. ***  Bleeding  Cramping  Nausea/vomiting  Thrombocytopenia  Fever/chills  All of the above  Don’t know |
| **SECTION 4: ATTITUDES**  **Q11 Please indicate your agreement with the following statements: ***  True  False  Unsure   1. I have the knowledge to counsel women about the process of medical abortion 2. I feel confident in managing medical abortions 3. I feel confident to assist in medical abortion provision 4. It is acceptable for nurse/nurse practitioners to be involved in assisting with provision of medical abortion 5. I think women need to know more about the availability of medical abortion |
| **Q12 Do you think the side effects of intrauterine devices outweigh the benefits? ***  Yes  Unsure  No  **Q13 Do you think the side effects of contraceptive implants outweigh the benefits? ***  Yes  Unsure  No  **Q14 What factors influence you to recommend long-acting reversible contraceptives to an eligible recipient? Please tick all that apply.** *****  Age  Cost  Patient’s BMI  Past history of abnormal cytology on Pap test / Cervical Screening Test  Past history of sexually transmitted infection  Marital status  History of abortions  History of full-term pregnancies  ☐Clinician preference  ☐Patient preference  I don’t recommend LARC  Other ________ |
| **Q15 What would dissuade you from assisting with a medical abortion to an eligible patient? Please tick all that apply. ***  I don't feel I have the knowledge to assist in the provision of a medical abortion  I don't feel I have the skills to assist in the provision of a medical abortion  Cost to patient  Cost to the clinic  Concerns about safety  Concerns about efficacy  I am afraid of the stigma associated with assisting in the provision of medical abortion  Lack of referral options to specialists/hospitals if a complication occurs  ☐ Previous negative experience supporting a patient undergoing medical abortion  I am a conscientious objector to termination of pregnancy  Other ________  None/NA  **Q16 What are the benefits of providing medical abortion in general practice? Please tick all that apply. ***  It is a cost-effective option for women  Opportunity to provide more comprehensive care  Offers continuity of care for women  Reduces women's need to travel to access an abortion service  Increased confidentiality for the woman  Contraceptive care can be discussed or provided at the same time  I can’t see any benefit  Other _____ |
| **SECTION 5: EXPERIENCES**  **Q17 Please indicate which option most closely reflects your experience***  Strongly disagree  Disagree  Neither  Agree  Strongly agree  NA  1.      The media take a balanced view on medical abortion  2.       I feel discriminated against by other healthcare professionals because of my decision to assist in medical abortion services  3.       I am proud that I work in abortion care  4.       I feel connected to others who do this work  5.       I feel that society appreciates the work I do in abortion care  6.       I am afraid that if I tell people I work in abortion care I could put myself or my loved ones at risk of harassment/violence  7.       I have never experienced harassment and/or violence as a result of working in abortion care  8.       I did not realise I had concerns assisting with medical abortions until I started working in the area  9.        I feel confident to assist with medical abortions. |

- Indicates mandatory questions
